# Expansion of Laboratory Capacity in the Eastern Mediterranean Region During the COVID‐19 Pandemic: Lessons Learned and Future Strategies for Sustainability

**DOI:** 10.1111/irv.70030

**Published:** 2024-11-04

**Authors:** John McCauley, Maria Van Kerkhove, Laith Jamal Abu Raddad, Luke Meredith, Richard Brennan, Abdinasir Abubakar, Amal Barakat

**Affiliations:** ^1^ Worldwide Influenza Centre (WIC) The Francis Crick Institute London UK; ^2^ Department of Epidemic and Pandemic Preparedness and Prevention World Health Organization Geneva Switzerland; ^3^ Infectious Disease Epidemiology Group, Weill Cornell Medicine‐Qatar Cornell University, Qatar Foundation–Education City Doha Qatar; ^4^ Eastern Mediterranean Regional Office World Health Organization Cairo Egypt; ^5^ Country Office for Lebanon World Health Organization Beirut Lebanon

**Keywords:** COVID‐19 pandemic, laboratory capacity, sustainability

The COVID‐19 pandemic posed unprecedented challenges to healthcare systems globally, necessitating a rapid and robust response from all sectors, from public health to commerce. A key effort in response was the need for a substantial expansion in laboratory testing and diagnosis to monitor the spread of the virus and to provide critical data to support effective public health measures. The scale of the threat drove research and innovation in laboratory diagnostics and genomic surveillance, enhancing testing capabilities and providing technological support to countries that previously did not have access to the key capabilities for rapid detection of pathogens that are necessary to prevent the next outbreak from becoming a pandemic.

The WHO Eastern Mediterranean Region (EMR) comprises the Occupied Palestinian Territories and 21 member states: Afghanistan, Bahrain, Djibouti, Egypt, Iran, Iraq, Jordan, Kuwait, Lebanon, Libya, Morocco, Oman, Pakistan, Qatar, Saudi Arabia, Somalia, Sudan, Syrian Arab Republic, Tunisia, United Arab Emirates, and Yemen. These countries have a diverse range of socio‐economic and demographic conditions, and many are facing humanitarian crises caused by civil conflict and natural disasters. Despite these challenges, member states in the region, supported by national, regional, and international stakeholders, were able to mount variable but largely robust laboratory responses, increasing COVID‐19 diagnostic and genomics capacity to covering 100% of the region [1]. This special issue provides an insight into the challenges faced in this rapid scale‐up of capacity, as well as the extraordinary efforts taken to overcome them during the pandemic.

The key initiatives highlighted throughout this issue are now being redirected towards a sustainable laboratory network with the capacity to detect and respond to new and re‐emerging pathogens that pose threats to public health in the region and globally, with the goal of preventing the next outbreak from becoming a pandemic. The investment in strengthening testing capacity, through molecular platforms such as PCR and genomics, that play a pivotal role in detecting and monitoring COVID‐19 and other respiratory viruses, are now being expanded to include detection of priority pathogens with epidemic and pandemic potential such as other respiratory pathogens (e.g., MERS‐CoV), arboviruses (e.g., dengue), and hemorrhagic fevers (e.g. CCHF), which periodically threaten the region. These capacities are being strengthened and sustained through the development of and investment in national and regional strategies to support genomic surveillance in the region, with efforts underway to establish steering committees and technical working groups to sustain, standardize and enhance genomic sequencing in the region [2], as well as the continued expansion of quality assurance networks to ensure that laboratories continue to produce robust, reliable results to support public health initiatives [3, 4].

The launch of the International Pathogen Surveillance Network (ISPN) is well timed with member states in the region to support in the sustainability of novel genomic and molecular testing platforms into the future. While next‐generation sequencing provides valuable insights into genomic characterization of pathogens and aids in the tracking of viral mutations and the identification of emerging SARS‐CoV‐2 variants, for example, the collective efforts need to focus on long‐term strategies to ensure that the platforms are sustainable financially and technologically. Indeed, the WHO Eastern Mediterranean Regional Office is working with member states and international stakeholders [2, 5], including IPSN, to implement a regional strategy for genomic surveillance, which will support and empower member states to develop genomic sequencing and capacity. This proactive approach will enable healthcare systems to adapt and tailor their response strategies in ways that maximize the impact of genomics, while minimizing the potential impact of future outbreaks.

Collaborative surveillance and knowledge‐sharing form the cornerstone of public health, with early detection, warning, and response being the key to preventing outbreaks from spreading. Key stakeholders in the region have been providing support for training in areas including laboratory protocols but focus now must turn to how to integrate these new platforms into existing surveillance networks to provide useable, epidemiologically relevant, and informative data that can support public health responses. Laboratories function as a key pillar of a public health response and need to work collaboratively with rapid response and surveillance teams to ensure that high quality samples and data are collected, to allow high quality results to be generated.

The investment in both research and development was also demonstrably helpful to the public health response in the region, ensuring that technological and infrastructure were deployed in the most useful way to provide diagnostic coverage to a broad population and to inform knowledge of the local outbreaks [6, 7, 8, 9, 10]. Information technology platforms developed during the pandemic supported the collation of patient data and subsequent dissemination of results, and these platforms can now be developed further to support data and sample management for a broad range of pathogens and disease states. Member states are now working with stakeholders to link these networks with existing surveillance platforms, to streamline data sharing across sectors as rapidly as possible [11, 12, 13]. The importance of streamlining and improving the capacity for data sharing cannot be understated, both in a multi‐sectoral fashion within a country and collaboratively across borders to ensure all countries are aware of challenges as they arise, and can mount appropriate, measured responses in a timely manner before a threat emerges, rather than during an emergency.

An often‐overlooked component of public health response is the investment in human resources. The COVID‐19 pandemic placed an extreme burden on health workers across the region, and the world, often working under extremely challenging conditions with the sustained pressure of samples continually needing urgent analysis [11]. This had a massive impact on staff wellbeing across the sector, and efforts need to be taken to ensure that laboratory and public health responses are sustainable and that the expertise gained from working under such conditions is not lost because of the pandemic slowing and investment reducing. Laboratories across the region took novel steps to support their staff, beyond wage investment, including areas such as staff mental health and physical well‐being, and will now look to apply the lessons learned to sustainable laboratory operations in future.

In conclusion, laboratory support and expansion in the Eastern Mediterranean Region during the COVID‐19 pandemic demonstrated the region's resilience and commitment to public health. By building on the lessons learned, sustaining investments in infrastructure, fostering collaboration, and prioritizing research and development, the Eastern Mediterranean Region can further strengthen its public health and healthcare systems to be better prepared to respond to future challenges. The COVID‐19 pandemic has highlighted the critical role of laboratories in disease surveillance, diagnosis, and research, emphasizing the need for continued support and investment in this vital sector. Through collective efforts, sustained commitment, and international engagement, the region can pave the way for a healthier and more resilient future for its population and at the same time inform global health responses.

## Author Contributions

**John McCauley:** conceptualization, writing – original draft, writing – review and editing. **Maria Van Kerkhove:** conceptualization, writing – original draft, writing – review and editing. **Laith Jamal Abu Raddad:** conceptualization, writing – original draft, writing – review and editing. **Luke Meredith:** conceptualization, writing – original draft, writing – review and editing. **Richard Brennan:** conceptualization, writing – original draft, writing – review and editing. **Abdinasir Abubakar:** conceptualization, writing – original draft, writing – review and editing. **Amal Barakat:** conceptualization, writing – original draft, writing – review and editing.

## Conflicts of Interest

The authors declare no conflicts of interest.

### Peer Review

The peer review history for this article is available at https://www.webofscience.com/api/gateway/wos/peer‐review/10.1111/irv.70030.

## Data Availability

The authors have nothing to report.
